# Incidence Density Rate of Neonatal Mortality and Predictors in Sub-Saharan Africa: A Systematic Review and Meta-Analysis

**DOI:** 10.1155/2020/3894026

**Published:** 2020-10-15

**Authors:** Zebenay Workneh Bitew, Ayinalem Alemu, Ermias Getaneh Ayele, Desalegn Abebaw Jember, Michael Tamene Haile, Teshager Worku

**Affiliations:** ^1^St. Paul's Hospital Millennium Medical College, Nursing Education Directorate, Addis Ababa, Ethiopia; ^2^Ethiopian Public Health Institute, Addis Ababa, Ethiopia; ^3^College of Health and Medical Sciences, School of Nursing and Midwifery, Haramaya University, Harar, Ethiopia

## Abstract

**Background:**

Neonatal mortality in Sub-Saharan countries is remarkably high. Though there are inconsistent studies about the incidence density rate of neonatal mortalities (IDR) and predictors in Sub-Saharan Africa, they are inconclusive to policymakers and program planners. In this study, the IDR of neonatal mortalities and predictors was determined.

**Methods:**

Electronic databases (Web of Science, PubMed, EMBASE (Elsevier), Scopus, CINAHL (EBSCOhost), World Cat, Google Scholar, and Google) were explored. 20 out of 818 studies were included in this study. The IDRs and predictors of neonatal mortality were computed from studies conducted in survival analysis. Fixed and random effect models were used to compute pooled estimates. Subgroup and sensitivity analyses were performed.

**Results:**

Neonates were followed for a total of 1,095,611 neonate-days; 67142 neonate-days for neonates treated in neonatal intensive care units and 1,028,469 neonate-days for community-based studies. The IDRs of neonatal mortalities in neonatal intensive care units and in the community were 24.53 and 1.21 per 1000 person-days, respectively. The IDRs of early and late neonatal mortalities neonatal intensive care units were 22.51 and 5.09 per 1000 neonate-days, respectively. Likewise, the IDRs of early and late neonatal mortalities in the community were 0.85 and 0.31, respectively. Not initiating breastfeeding within one hour, multiple births, rural residence, maternal illness, low Apgar score, being preterm, sepsis, asphyxia, and respiratory distress syndrome were independent predictors of time to neonatal mortality in neonatal intensive care units and male gender, perceived small size, multiple births, and ANC were predictors of neonatal mortality in the community.

**Conclusion:**

The incidence density rate of neonatal mortality in Sub-Saharan Africa is significantly high. Multiple factors (neonatal and maternal) were found to be independent predictors. Strategies must be designed to address these predictors, and prospective studies could reveal other possible factors of neonatal mortalities.

## 1. Background

Neonatal mortality rate (NMR) is defined as the death of a newborn within 28 days of birth, and it is expressed per 1000 live births [1]. It is also classified as early (the first 7 days) and late (7 to 28 days) neonatal mortality rates [2, 3]. It is the most vulnerable period in which around one million newborns die in the early neonatal period and 2.8 million die in the late neonatal period [4]. Neonatal mortality rate in the least developed countries (26 per 1000 live births) is significantly lower than NMR in the globe (19 per 1000 live births) [5]. In Sub-Saharan Africa (SSA), NMR was found to be 29 per 1000 live births that accounted for 36% of under-five mortalities [6].

Neonatal mortality (NM) is a major public health problem that endangers the survival of children with a remarkable variation between developed (4 to 46%) and developing countries (0.2 to 64.4%) [7]. It contributes to 44% of under-five mortalities throughout the world and more than 99% of NM took place in low- and middle-income countries, including SSA [2, 3, 8], with a slow progress in decreasing of NM being seen in African and Asian countries [9]. The highest incidence of neonatal mortality was also reported in Sub-Saharan countries, and the burden remained high even after the implementation of the millennium development goals. For instance, NMR in Ethiopia remained stagnant for decades with the current NMR of 30 per 1000 live births [10].

Even though the burden of NMR is considerably high in SSA, there are very few inconsistent studies about the incidence dentistry rate (IDR) of NM. The predictors of time to NM are also very limited. The IDR of NM in SSA ranged from 1.00 per 1000 neonate-days [11] to 39.04 per 1000 neonate-days [12] in Ethiopia and from 1.93 [13] to 30 [14] per 1000 neonate-days in the other Sub-Saharan countries. The burden of IDR of NM is significantly higher in the studies conducted among neonates who were treated in the neonatal intensive care units (NICUs) [12–22] of SSA as compared with community-based studies [11, 23, 24]. The predictors of time to NM were reported by very limited studies that can be classified as service-related (type of delivery, place of delivery, antenatal and postnatal cares, immunization services, and physical distances of health institutions), maternal-related (age, multiple pregnancy, residence, initiation of breast feeding within one hour of birth, delivery complications, and maternal illness), and newborn-related factors (vital signs, low birth weight, gender, and comorbidities like sepsis, hypothermia, asphyxia, respiratory distress syndrome, and jaundice) [11–13, 15–23, 25–30].

All these studies revealed that the IDR of NM remained unacceptably high in SSA. The findings are also inconsistent and inconclusive to policymakers and stakeholders due to variability among the findings. Thus, the pooled IDR of NM and its predictors in SSA was determined in this systematic review and meta-analysis. As to our knowledge, this study is the first of its type to identify the predictors of time to NM using factors that were reported in hazard ratios (HRs). In the original studies, HRs were computed using survival analysis model. The pooled IDRs and predictors were computed for both studies conducted in the NICUs and community-based studies. Hence, the findings of this study will have a vital implication for policymakers, program planners, and to the scientific community. The findings will also have a special importance of depicting gaps hindering the progress of countries towards achieving the sustainable development goals (SDGs), which has a plan of ending preventable causes deaths of newborns by all countries with an aim of reducing neonatal mortality to as low as 12 per 1000 live births by 2030 [31].

## 2. Methods

### 2.1. Eligibility Criteria and Information Sources

In this systematic review and meta-analysis, studies conducted in SSA with an objective of assessing incidence density rate (IDR) of neonatal mortality and predictors were included. The studies were assessed using study area, study setups, title, abstract, and full texts prior to inclusion in this study. This study is prepared based on preferred reporting items for systematic reviews and meta-analysis (PRISMA) statement [32]. In the current study, published articles, surveys, and unpublished article that were conducted in English language were explored and included accordingly. Cohort studies that were conducted in clinical and community-based setups reporting the incidence density rates of neonatal mortality and predictors using a hazard function were included in this meta-analysis. Studies that were conducted from inception to June 2020 were searched. However, studies that did not report either IDR of the predictors of neonatal mortality based on the principles of survival analysis or hazard functions were not included in the current meta-analysis. The EndNote X8 reference manager was used to manage retrieved articles.

### 2.2. Search Strategy and Selection of Studies

Comprehensive searching was conducted to identify the studies. Electronic databases, grey literature sources, and reference lists of articles were explored by three investigators (ZWB, AA, and TW), independently. Web of Science, PubMed, EMBASE (Elsevier), Scopus, CINAHL (EBSCOhost), World Cat, Google Scholar, and Google were explored to find out articles. Searching was conducted using key terms: (a) population (neonates, newborns, and infants), (b) exposure (associated factors, risk factors, determinants, and predictors), (c) outcome (incidence density rate of neonatal mortality, death, mortality, time to death, survival status, and treatment outcomes), (d) study design (cohort studies, epidemiology, observational, and national demographic and health surveys), (e) study setting (hospitals, neonatal intensive care unit, NICU, community-based surveys, and health centers), and (f) location (Sub-Saharan Africa, SSA, and Sub-Saharan countries). The Boolean search operators such as “OR” and “AND” were used during the searching process. Key terms were verified for appropriateness prior to actual searching. Example of search strategy in PubMed: ((((((((((((((((Incidence density rate) AND (“Infant, Newborn”[Mesh])) AND (“Mortality”[Mesh])) OR (“Death”[Mesh])) OR (Time to death)) OR (survival status)) OR (“Treatment Outcome”[Mesh]) AND (predictors))) OR (Determinants)) OR (Associated factors)) OR (“Risk Factors”[Mesh])) AND (“Intensive Care Units, Neonatal”[Mesh])) AND (“Africa South of the Sahara”[Mesh]))).

### 2.3. Data Extraction Process

Structured and pretested data extraction checklist was used to collect information by three authors (ZWB, DAJ, and EGA). Name of the author(s), publication year, study country, sample sizes, study population, treatment outcome, type of neonatal mortality, follow-up in neonate-days, the incidence density rate of death, and factor in HRs were extracted for this study. During the extraction process, disagreements between the three were solved by the other author (MTH). The data for subgroup analysis based on the type neonatal mortalities and the HRs of the studies were extracted from the included studies. The appropriateness of each datum was verified before the analyses (*Additional file 1*).

### 2.4. Quality Assessment of Studies

The qualities of the included studies were independently appraised by two authors (ZWB and MTH) using Joanna Briggs Institute (JBI) Critical Appraisal Checklist for Cohort Studies [33]. The tool has Yes, No, Unknown, and Not Applicable options; 1 is given for Yes and 0 for other options. The minimum score was 0, and 11 was the maximum one. The scores were summed up and changed to percentages. Studies with >50% quality scores were included in this meta-analysis *(Additional file 2)*. The mean scores of the two reviewers were used for the final decision of inclusion of the studies in this systematic review and meta-analysis. During critical appraisal, the third author (TW) participated actively in solving disagreements between two authors.

### 2.5. Summary Measures

The primary outcome of this study was the incidence density rate of neonatal mortality in SSA in both neonatal intensive care units and in the community setups. The IDR was also computed for early neonatal mortality (birth to 6 days) and late neonatal mortality (7 to 28 days). Time to neonatal mortality was calculated from admission to NICU to death or discharge and from birth to death (for community-based studies). Neonates were followed in days, and the IDRs were calculated by dividing the number of neonates died to total follow-up periods in person-days (neonate-days) and multiplying it by 1000. The IDRs were presented per 1000 per person-days (neonate-days). The second outcomes were predictors of time to neonatal mortality that were computed from studies reporting predictors in the form of HRs. The pooled HRs determining time to death were computed using the crude hazard ratios with 95% confidence intervals (CI) that were reported in the original studies. This was done to control confounders. STATA (version 15) software was used to compute the pooled estimates. The “metan” command was used to compute the pooled hazards ratios of each predictor variable. The pooled hazard ratios (PHRs) were presented with 95% CI. Independent predictors were declared when the 95% CI did not include one. The effect sizes were IDR of neonatal mortality and hazard ratios predicting time to neonatal mortality.

### 2.6. Statistical Methods and Analysis

In the present meta-analysis, STATA Version 15 (STATA Corporation, College Station Texas) software was used for computing the pooled estimates of both IDR of neonatal mortality and predictors of NM. The pooled estimates were computed using both random and fixed effect models. Results were presented by using either the fixed (when there is no significant heterogeneity among studies) and random effect models (when there is heterogeneity among studies). Subgroup analyses were performed using the type of neonatal mortality (early NM or late NM), study setting (NICU or community based), and country the original studies were conducted. The predictors of neonatal mortality were identified for both clinical-based and community-based studies. The pooled estimates of predictors were also presented separately. The pooled estimates were presented with 95% CI. Meta-analyses were presented using forest plot, summery tables, and texts.

### 2.7. Publication Bias and Heterogeneity

Publication bias was assessed by looking at the asymmetry of the funnel plot and/or statistical significance of Egger's regression test. Publication bias was declared when Egger's regression test was significant (*p* < 0.05) [34]. Heterogeneities among studies were explored using forest plots, *I*^2^ test, and Cochrane *Q* statistics [35]. The *I*^2^ values of 25%, 50%, and 75% were interpreted as low, medium, and high heterogeneity, respectively [36]. In this study, the presence of heterogeneity was declared and justified when *I*^2^ ≥ 50% with *p* value < 0.05. The sources of possible significant heterogeneities were explored through subgroup analyses and sensitivity analysis (using Duval and Tweedie's Trim and Fill analysis in the random effect model).

## 3. Results

### 3.1. Selection of Eligible Studies

**Figure 1:**
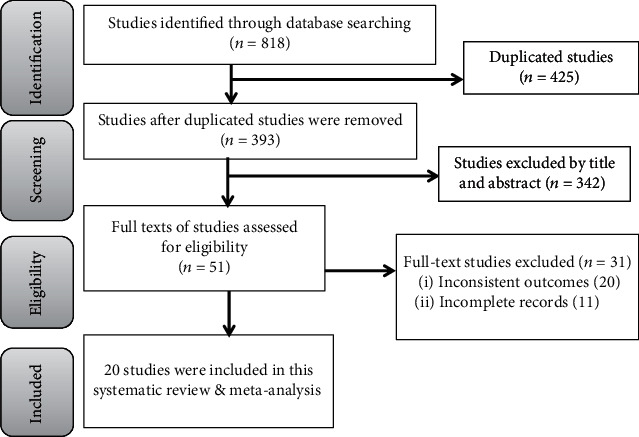
PRISMA flow chart showing the literature search process.

In the initial search, 818 studies were found. These studies were retrieved from electronic databases and other sources. Of these studies, 425 were duplicate files and 342 studies were removed after screening using titles and abstracts. The full texts of 51 studies were reviewed. Finally, 20 studies [11–30] were included in the final analysis of this systematic review and meta-analysis (Figure 1).

### 3.2. Characteristics of the Studies

All studies included in this study were cohort studies. Regarding the study setting, 12 studies [12–22, 28] were conducted in NICUs and three [11, 23, 24] were community-based prospective cohort studies. The others [25–27, 29, 30] were reported from retrospective analysis of demographic and health surveys of the respective countries. From the included studies to this systematic review and meta-analysis, the majorities (15 articles) [11, 12, 15–24, 28–30] were conducted in Ethiopia, three were performed in Nigeria [25–27], and others were done in Burkina Faso [13] and Uganda [14]. In this study, neonates were followed in days and the follow-up times were reported in neonate-days. Neonates were followed for a total of 1,095,611 neonate days, of which neonates admitted in NICUs of SSA were followed for 67142 neonate-days and neonates in community-based studies were followed for a total of 1,028,469 neonate-days. The IDR of NM in NICU ranged from 1.93 per 1000 neonate-days [13] to 39.04 per 1000 neonate days [12]. Likewise, it ranged from 1.00 [11] to 1.51 [23] per 1000 neonate-days in the community-based studies (Table 1).

In the current study, the predictors of time to NM were presented in the form of hazard ratios. Out of 20 articles included in this systematic review and meta-analysis, 11 articles [12, 13, 15–22, 28] reported the predictors of time to NM among neonates admitted to NICU. Similarly, the predictors of time to NM were reported by seven [11, 23, 25–27, 29, 30] community-based studies, studies conducted through the analysis of secondary data obtained from demographic and health surveys. Regarding the quality scores of articles, nine [11, 15–21, 23], eight [12, 14, 22, 24–26, 28, 30], and three [13, 27, 29] were classified under high, medium, and low qualities, respectively.

### 3.3. The Pooled Incidence Rate of Neonatal Mortality in Sub-Saharan Africa

**Figure 2:**
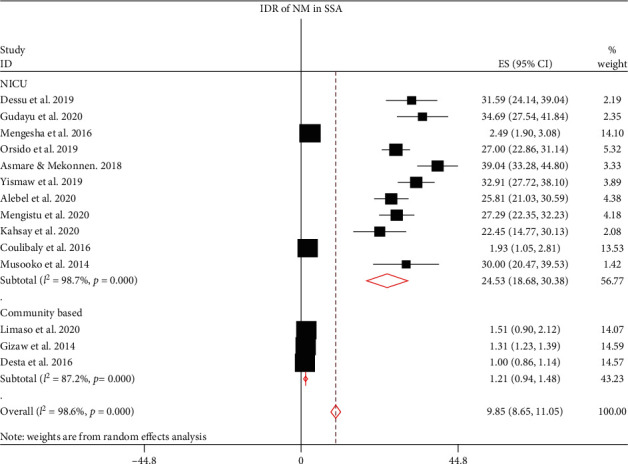
The incidence density rate of neonatal mortality in SSA.

Fourteen articles were included to estimate the pooled IDR of NM in SSA. Of these, 11 articles [12–22] were used to compute the pooled IDR of NM among neonates admitted to NICU. Three articles [11, 23, 24] were included to estimate the pooled IDR of NM in the community. The pooled IDR of NM in SSA was found to be 9.85 per 1000 neonate-days (95% CI: 8.65, 11.05; *I*^2^ = 98.6%, *p* ≤ 0.001). The IDR of NM among neonates admitted to NICUs of SSA was 24.53 per 1000 person-days (95% CI: 18.68, 30.38; *I*^2^ = 98.7%, *p* ≤ 0.001), whereas the pooled IDR of NM in the community was 1.21 per 1000 person-days (95% CI: 0.94, 1.48; *I*^2^ = 87.2%, *p* ≤ 0.001) in the random effect model (Figure 2).

### 3.4. Subgroup Analysis of Incidence Rate of Neonatal Mortality in Sub-Saharan Africa

**Figure 3:**
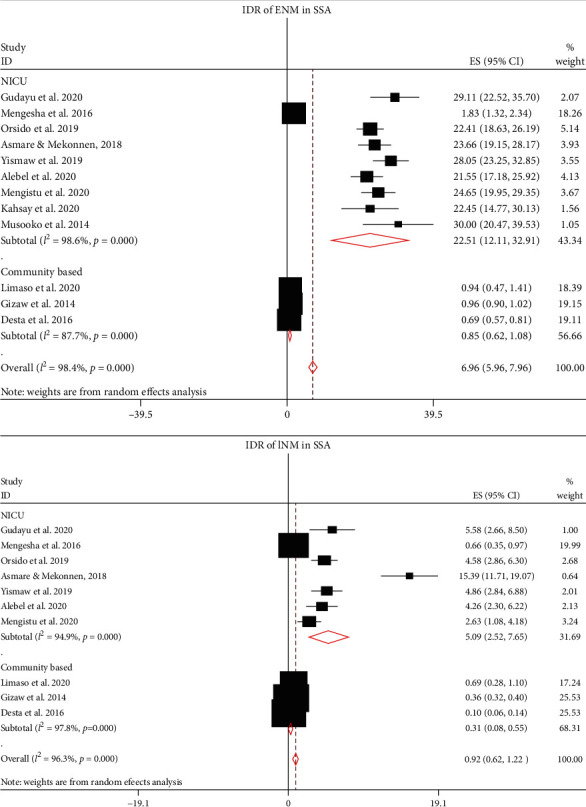
The IDR of early neonatal mortality (ENM) and late neonatal mortality (LNM) in SSA.

Due to the presence of higher heterogeneity both in the clinical-based and community-based studies, subgroup analysis was done on the type on NM (ENM and LNM). Twelve studies were eligible to estimate the pooled IDR of ENM in SSA, of which nine [12, 14, 16–22] were used to compute IDR in NICU and three [11, 23, 24] were used for community-based studies. The overall pooled IDR of ENM in SSA was 6.96 per 1000 neonate-days (95% CI: 5.96, 7.96; *I*^2^ = 98.4%, *p* ≤ 0.001), of which IDR of ENM in NICU was 22.51 per 1000 neonate-days (95% CI: 12.11, 32.91; *I*^2^ = 98.6%, *p* ≤ 0.001) and 0.85 per 1000 neonate-days (95% CI; 0.62, 1.08; *I*^2^ = 87.7%, *p* ≤ 0.001) in the community. Likewise, the pooled IDR of LNM (0.92 per1000 neonate-days; 95% CI: 0.62, 1.22; *I*^2^ = 96.3%, *p* ≤ 0.001) was computed using 10 eligible articles. Of these, the IDR of LNM in NICU was 5.09 per 1000 neonate-days (95% CI: 2.52, 7.65; *I*^2^ = 94.9%, *p* ≤ 0.001) that was computed from seven eligible studies [12, 16–21]. The IDR of LNM in the community [11, 23, 24] was found to be 0.31 per 1000 neonate-days (95% CI: 0.08, 0.55; *I*^2^ = 97.8%, *p* ≤ 0.001) (Figure 3).

**Figure 4:**
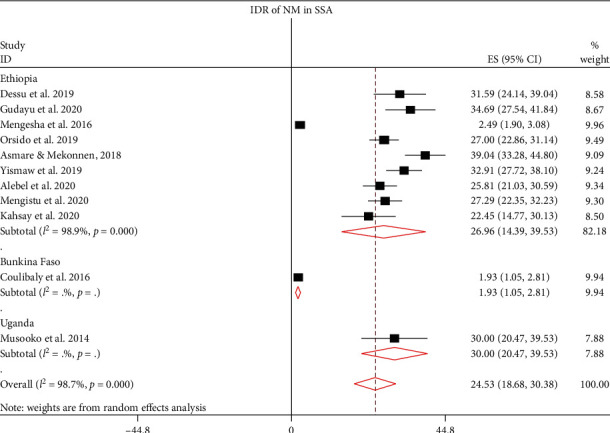
The IDR of neonatal mortality among neonates treated in the NICUs of SSA.

**Figure 5:**
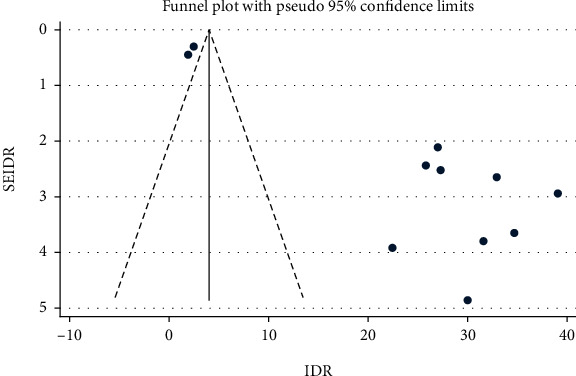
Funnel plot showing publication bias among studies used to compute the IDR of NM among neonates treated in the NICUs of SSA.

**Figure 6:**
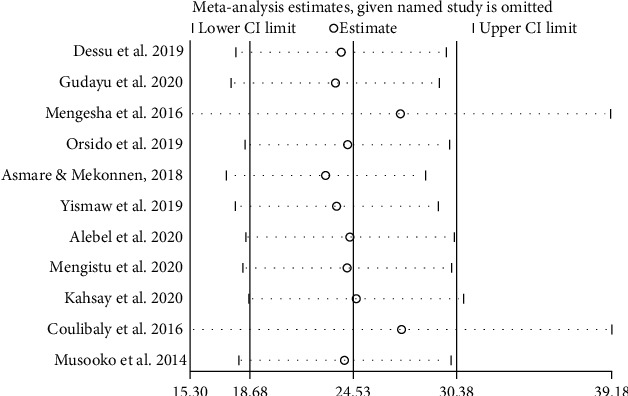
Sensitivity analysis for studies included to estimate the pooled incidence density rate of neonatal mortality in NICUs of SSA.

The IDR of NM among neonates admitted to NICUs in SSA was further analyzed based on countries, where the original studies were performed. This was done due to significant heterogeneities among the included studies and to verify the possible source of heterogeneities. Hence, the IDR of NM in Ethiopia, Burkina Faso, and Uganda were 26.96 (95% CI: 14.39, 39.53; *I*^2^ = 98.9%, *p* ≤ 0.001), 1.93 (95% CI: 1.05, 2.81), and 30.00 (95% CI: 20.47, 39.53) per 1000 neonate-days, respectively (Figure 4). However, heterogeneity among studies remained higher and the possibility of its association with publication bias was explored using funnel plot and Egger's regression test. The funnel plot revealed that there was publication bias, and the asymmetry of funnel plot was confirmed by a significant Egger's test (*p* ≤ 0.001) (Figure 5). Finally, sensitivity analysis was done and it was found that no single study affected the pooled IDR. However two studies [13, 17] could have a slight contribution to heterogeneity among the studies used to compute the pooled IDR of NM among neonates treated with in NICUs of countries in Sub-Saharan Africa (Figure 6).

### 3.5. Predictors of Neonatal Mortality in Sub-Saharan Africa

The predictors of time to NM were computed for both studies conducted in NICUs and studies conducted through analysis of secondary data (community-based studies). For a predictor to be included in this meta-analysis, it has to be statistically significant at least in two studies. Eleven studies [12, 13, 15–22, 28] were included in the analysis of the predictors of time to NM among neonates treated in NICUs of SSA. Fifteen predictors were included in this meta-analysis: low Apgar score, not initiating breastfeeding within one hour of birth, multiple births, CS delivery, home delivery, low birth weight, being preterm, being female, no ANC follow-up, rural residence, sepsis, asphyxia, respiratory distress syndrome (RDS), hypothermia, and maternal illness. Of these CS delivery, home delivery, low birth weight, being female, no ANC follow-up, and hypothermia were found to be statistically insignificant. Low Apgar score, not breastfeeding within one hour of birth, multiple births, being preterm, rural residence, sepsis, asphyxia, RDS, and maternal illness were independent predictors of time to NM. Neonates with low first and 5^th^ minute Agar score were 3.47 (PHR: 3.47, 95% CI: 2.53, 4.41) times more likely to die as compared to neonates who were born with normal Apgar score. The hazard of death of neonates who did not initiate breastfeeding within one hour of birth was 3.77 (PHR: 3.77, 95% CI: 1.44, 6.10) times than the counterparts. Neonates who were part of multiple births were four times (PHR: 3.92, 95% CI: 2.64, 5.37) more likely to die compared to neonates born from single pregnancies. Likewise, preterm neonates were 3.78 (PHR: 3.78, 95% CI: 2.28, 5.27) times more likely to die than term neonates. Neonates who were from a rural residence were nearly two times (PHR: 1.91, 95% CI: 1.30, 2.52) more likely to die than neonates who were from urban residence. The likelihood of death of neonates whose mother had a history of illness during pregnancy was higher than the counterparts (PHR: 1.91, 95% CI: 1.41, 2.41). Moreover, comorbidities (sepsis, asphyxia, and RDS) increased the hazards of death of neonates who were treated in the NICUs of SSA. The hazard of death of neonates with sepsis was 1.94 (PHR: 1.94, 95% CI: 1.53, 2.34) times than neonates free from sepsis. Similarly, the likelihood of death among neonates with perinatal asphyxia were two (PHR: 2.03, 95% CI: 1.32, 2.75) times as compared to neonates who had no perinatal asphyxia. Besides, neonates who had RDS were 2.49 (PHR: 2.49, 95% CI: 1.98, 3.00) times more likely to die than neonates who did not have RDS (Table 2).

In the current study, seven community-based studies [11, 23, 25–27, 29, 30] were included to compute the PHRs predicting time to death of neonates in the community. From the eligible predictors, three variables (home delivery, CS delivery, and pregnancy complications) were not significantly associated with time to NM, whereas male gender, perceived small size, multiple births, and ANC follow-up were significantly associated with time to NM. Perceived small size is a substitute of low birth weight in NICU-based studies due to the fact that birth weights and weeks of delivery are usually unclear in the community-based studies. Male neonates were 1.31 (PHR: 1.31, 95% CI: 1.22, 1.41) times more likely to die than females. Neonates who were perceived as small in size by their mother were 1.85 (PHR: 1.85, 95% CI: 1.05, 2.65) times highly likely to die as compared to neonates who were perceived to be normal in size at birth. Similarly, multiple births increased the likelihood of death by 3.71 (PHR: 3.71, 95% CI 1.72, 5.69) times than single births. Finally, this study revealed that ANC follow-up could decrease the hazard NM by 12% (PHR: 0.82, 95% CI: 0.66, 0.97). Only one study [23] reported that no postnatal care increased the likelihood of neonatal mortality in the community (HR: 2.80, 95% CI: 1.16, 6.75) (Table 2).

## 4. Discussion

This study is the first of its type in investigating the pooled incidence density rate of neonatal mortality and its predictors among neonate treated in the neonatal intensive care units and community setups of SSA. Though there are limited studies in SSA about IDR of NM and its predictors, 20 studies were found to fulfill the eligibility criteria of this study. Thus, 14 studies were used to compute the pooled IDR of NM and 18 studies were used to identify the predictors of NM both in the clinical setups and in the community.

The pooled IDRs of NM were computed using fixed and random effect models. However, the final estimates were reported using random effect model, due to the presence of remarkable heterogeneity among the included studies (*I*^2^ > 90%, *p* ≤ 0.001). The pooled IDR of NM in SSA is 9.85 per 1000 neonate-days, of which the IDR of neonates who were treated in NICUs is 24.53 per 1000 person-days and 1.21 per 1000 person-days in the community. Due to high heterogeneity among studies, these pooled estimates were presented with a random effect model. In this study, subgroup analyses were performed to compute the pooled IDR of NM. Based on the type of NM, IDR of ENM is 6.96 per 1000 neonate-days, of which IDR of ENM in NICU is 22.51 per 1000 neonate-days and 0.85 per 1000 neonate-days in the community. Likewise, the IDR of LNM is 0.92 per1000 neonate-days. Of these, IDR in NICU is 5.09 per 1000 neonate-days and the IDR of LNM is 0.31 per 1000 neonate-days. The IDR of NM in NICUs of SSA was further analyzed based on study country. The IDR of NM in Uganda, Ethiopia, and Burkina Faso were 30.00, 26.96, and 1.93 per 1000 neonate-days, respectively. Finally, trim and fill analysis (sensitivity analysis) was performed; however, no significant change was observed in the pooled estimates and heterogeneities between the studies included in this meta-analysis. The possible source of heterogeneity could be best explained by the presence of a wide variation in NM among Sub-Saharan countries that might be related to differences in the quality of healthcare governance, prevalence of HIV, and socioeconomic deprivation [37]. From these findings, it can be deduced that the IDR of NM in most of studies in NICUs is significantly higher than IDR in the community. The pooled IDRs are also remarkably higher NICUs. This could be attributed to the high burden of comorbidities in neonates admitted to NICUs, and most of neonates admitted to NICUs are preterm newborns which could uplift the likelihood of NM [38].

Regarding the predictors of NM, the predictors were classified into two. The first were those predicting time to NM of neonates who were treated in the NICUs of SSA. The second were predictors that determine the time to NM of neonates from birth to death using community-based studies. The independent predictors of NM among neonates who were treated in NICUs were further classified as maternal-related and newborn-related factors. The maternal-related factors were not initiating breastfeeding within one hour of birth, multiple births, rural residence, and maternal illness. The likelihood of NM during delayed initiation of breastfeeding is significantly higher as compared to the early initiation (with in 1 hour of birth) and this is supported by previous meta-analyses [39–41]. Similarly, the hazard of death among neonates born from multiple pregnancies is considerably higher than single pregnancies. The possible elucidation for this is that multiple births are usually associated with preterm births that can amplify the risk of NM [42, 43]. Rural residence is also an independent predictor of NM and the possible rational might be lack of access to health care services during antenatal and postnatal periods which could increase NM. Besides, maternal illness during pregnancy is found to have a significant association with NM and this is supported by a previous study finding [44].

The newborn-related factors were low Apgar score in the first and fifth minute of birth, being preterm, sepsis, asphyxia, and RDS. Low Apgar score in the first and 5^th^ minute of birth is found to increase the likelihood of NM, and it is corroborated by a previous finding that depicted low Apgar scores have a higher predictive value of NM [45]. The hazard of death of preterm babies is remarkably higher than neonates born on the due date. This could be due to the fact that preterm newborns are at greater risk of death, which could be resulted from physical and physiologic immaturities [46, 47]. In addition, comorbidities (sepsis, asphyxia, and RDS) increased the hazards on NM. It is clear that neonatal sepsis is one of the public health problems affecting the survival of children, primarily in countries included in this study [48]. Neonatal sepsis is frequently reported by prior studies as a predictor of NM [2, 49, 50]. Likewise, perinatal asphyxia is found to have a main predictor of NM. This could be due the fact that asphyxia is caused by interruption of blood flow to the placenta that could further decrease blood flow to the vital organs like the kidney, brain, heart, and lung [51, 52]. Another possible explanation for this is that the presence of high prevalence of perinatal asphyxia in East and Central Africa could be associated with increased incidence of NM in SSA [53]. Respiratory distress syndrome is also found to increase the hazard of NM of neonates treated in the NICUs of SSA. The mechanism of NM secondary to RDS is nearly similar with asphyxia, and RDS is the main predictor of NM which is supported by other findings [54, 55].

In the current study, the predictors of NM among neonates born in SSA were male gender, perceived small size, multiple births, and ANC follow-up. These predictors were computed from demographic and health surveys of the respective countries. The likelihood of NM in males is significantly higher than females. This could be associated with physiologic or hormonal differences, and the other possible rationales need to be explained by future researches. Likewise, perceived small size as reported by mothers of neonates is significantly associated with NM. This could be due to the fact that it is highly likely for perceived small sized babies to be either low birth weight or preterm and the likelihood of NM is highly likely in either of the two cases [56]. Multiple births are also independent predictors of NM in the community-based studies similar to studies conducted in the NICUs. In the present study, access to antenatal care is another independent determinate of prohibiting death neonates in SSA. This is supported a prior meta-analytic finding which revealed utilization of at least one antenatal visit could decrease NM by up to 39% [57]. Besides, only one study reported that no postnatal follow-up increased the hazard of NM [23]. There were very limited studies about the effect of postnatal care on the survival of newborns in SSA and it is a potential area of investigation.

In general, the findings of this study pinpointed that NM in SSA is caused by multiple predators, which are informative to program planners and policymakers of perinatal cares. The policymakers need to focus on promotion breastfeeding in the first one hour of birth. The NICUs should be organized to the standard. This can mitigate the three leading causes (asphyxia, preterm-related complications, and sepsis) NM in SSA. Besides, designing policies focusing on universal access of antenatal and postnatal cares may minimize the maternal causes of NM, such as maternal illnesses in SSA.

### 4.1. Strengths and Limitations

The main strength of this study is that the reputable databases were explored to find all possible articles. This study is the first of its type in determining the IDR of NM in SSA and unfolding the possible predictors of NM using hazard ratios. The predictors were separately estimated from studies in NICUs and community-based studies. This finding will have a paramount importance for program planners and policymakers in SSA, where the burden of NM is considerably high. Despite these strengths, the presence of scant studies in countries other than Ethiopia could obscure some other predictors of time to NM in SSA. Lack of reports on IDR of NM in community-based studies could also be associated with under estimation of IDR of NM in SSA. Prospective cohort studies in countries other than Ethiopia and community-based prospective cohort studies could unfold all possible causes of NM in the future.

## 5. Conclusion

The incidence density rate of neonatal mortality among neonates treated in neonatal intensive care units of SSA is significantly high. It is also considerable in the community. The hazard of neonatal mortality in NICUs is increased by comorbidities (sepsis, asphyxia, and RDS), low Apgar scores, being preterm, not initiating breastfeeding within one hour of birth, multiple births, rural residence, and maternal illness. Likewise, male gender, perceived small size, multiple births, and ANC follow-up were independent predictors of time NM in the communities of SSA. Designing strategies to address all these predictors could decrease the high burden of NM in SSA. We also recommend prospective longitudinal researches to be done in the respective Sub-Saharan countries to identify all possible bottlenecks of neonatal survival in SSA.

## Figures and Tables

**Table 1:** Incidence density rates (IDRs) of neonatal mortality in Sub-Saharan Africa.

Author year	Country	Sample size	Study population		Treatment outcome		Type of NM		Neonate days	IDR/1000 neonate days (95% CI)	Quality score
			Preterm	Term	Dead	Censored	ENM	LNM			
Dessu et al., 2019 [15]	Ethiopia	332	37	30	67	265	—	—	2121	31.59 (24.14, 39.04)	High
Gudayu et al., 2020 [16]	Ethiopia	504	—	—	87	417	73	14	2508	34.69 (27.54, 41.84)	High
Kayode et al., 2016 [37]	Ethiopia	1152	—	—	68	1084	50	18	27357.5	2.49 (1.90, 3.08)	High
Orsido et al., 2019 [18]	Ethiopia	964	84	75	159	804	132	27	5889	27.00 (22.86, 31.14)	High
Asmare, 2018 [12]	Ethiopia	571	170	—	170	401	103	67	4354	39.04 (33.28, 44.80)	Medium
Yismaw et al., 2019 [19]	Ethiopia	516	149	—	149	—	127	22	4527	32.91 (27.72, 38.10)	High
Alebel et al., 2020 [20]	Ethiopia	513	42	67	109	404	91	18	4223	25.81 (21.03, 30.59)	High
Mengistu et al., 2020 [21]	Ethiopia	612	69	45	114	498	103	11	4177.8	27.29 (22.35, 32.23)	High
Kahsay et al., 2020 [22]	Ethiopia	253	—	—	32	221	32	—	1425.32	22.45 (14.77, 30.13	Medium
Coulibaly et al., 2016 [13]	Burkina Faso	341	—	—	18	323	—	—	9326.4	1.93 (1.05, 2.81)	Low
Musooko et al., 2014 [14]	Uganda	341	—	—	37	304	37	—	1233.3	30.00 (20.47, 39.53)	Medium
Wosenu et al., 2017 [28]	Ethiopia	490	—	—	171	319	171	—	—	—	Medium
Limaso et al., 2020 [23]	Ethiopia	584	—	584	24	—	15	11	15894	1.51 (0.90, 2.12)	High
Gizaw et al., 2014 [24]	Ethiopia	—	—	—	1055	—	768	287	803370	1.31 (1.23, 1.39)	Medium
Desta et al., 2016 [11]	Ethiopia	7367	—	—	209	7158	144	20	209205	1.00 (0.86, 1.14)	High
Wakgari and Wencheko, 2013 [30]	Ethiopia	8651	—	—	517	—	367	150	—	—	Medium
Ezeh et al., 2014 [26]	Nigeria	27147	—	—	996	26151	—	—	—	—	Medium
Dahiru, 2017 [27]	Nigeria	244836	—	—	8176	236660	8176	—	—	—	Low
Dahiru, 2015 [25]	Nigeria	119024	—	—	3772	115,252	3772	—	—	—	Medium
Mekonen et al., 2013 [29]	Ethiopia	32 042	—	—	—	—	—	—	—	—	Low

**Table 2:** Predictors of neonatal mortality in Sub-Saharan Africa.

Predictors of neonatal mortality among neonates treated in the neonatal intensive care units (NICUs) of SSA.				
Predictors	Included studies	HR (95% CI)	Pooled HR (95% CI)	Heterogeneity
Low Apgar score	Dessu et al., 2019	4.8 (3.61, 9.87)	3.47 (2.53, 4.41)^∗^	*I* ^2^ = 0.0%, *p* = 0.603
	Asmare & Mekonnen, 2018	3.83 (2.70, 5.44)		
	Alebel et al., 2020	2.70 (1.50, 4.80)		
	Kahsay et al., 2020	3.13 (1.43, 6.86)		
No BF in 1 hour	Dessu et al., 2019	1.79 (1.09, 2.96)	3.77 (1.44, 6.10)^∗^	*I* ^2^ = 85.0%, *p* ≤ 0.001
	Mengesha et al., 2016	21.63 (12.82, 36.49)		
	Orsido et al., 2019	7.42 (4.73, 11.64)		
	Alebel et al., 2020	2.00 (1.30, 3.20		
Multiple births	Dessu et al., 2019	2.40 (1.19, 4.93	3.92 (2.64, 5.37)^∗^	*I* ^2^ = 58.0%, *p* = 0.068
	Orsido et al., 2019	4.43 (3.24, 6.07)		
	Mengistu et al., 2020	5.68 (2.95, 6.73)		
	Kahsay et al., 2020	2.73 (1.18, 6.32)		
CS delivery	Dessu et al., 2019	1.90 (1.08, 3.34)	0.99 (0.04, 1.95)	*I* ^2^ = 85.0%, *p* ≤ 0.001
	Orsido et al., 2019	0.26 (0.15, 0.46)		
	Mengesha et al., 2016	1.17 (0.65, 2.10)		
Home delivery	Gudayu et al., 2020	2.26 (1.04, 4.90)	2.19 (0.97, 3.410)	*I* ^2^ = 0.0%, *p* = 0.925
	Yismaw et al., 2019	2.14 (1.08, 4.23)		
Low birth weight	Mengesha et al., 2016	11.49 (4.49, 29.39)	4.85 (0.68, 10.37)	*I* ^2^ = 65.4%, *p* = 0.056
	Kahsay et al., 2020	7.30 (3.41, 15.63)		
	Yismaw et al., 2019	1.56 (1.10, 2.21)		
Preterm	Gudayu et al., 2020	2.55 (1.59, 4.08)	3.78 (2.28, 5.27)^∗^	*I* ^2^ = 49.3%, *p* = 0.095
	Orsido et al., 2019	3.41 (2.46, 4.73)		
	Asmare & Mekonnen, 2018	6.31 (3.89, 10.24)		
	Kahsay et al., 2020	5.83 (2.52, 13.49)		
	Coulibaly et al., 2016	17.60 (6.90, 44.8)		
Female gender	Orsido et al., 2019	0.59 (0.42, 0.84)	0.87 (0.32, 1.41)	*I* ^2^ = 82.6%, *p* = 0.003
	Mengistu et al., 2020	1.55 (0.96, 2.01)		
	Kahsay et al., 2020	0.56 (0.26, 1.19)		
No ANC follow-up	Orsido et al., 2019	12.96 (7.86, 21.39)	7.24 (-2.86, 17.33)	*I* ^2^ = 88.3%, *p* = 0.003
	Alebel et al., 2020	2.60 (1.50, 4.60)		
Rural residence	Mengistu et al., 2020	1.98 (1.27, 2.66)	1.91 (1.30, 2.52)^∗^	*I* ^2^ = 0.0%, *p* = 0.683
	Kahsay et al., 2020	1.68 (0.84, 3.36)		
Sepsis	Gudayu et al., 2020	1.95 (1.06, 3.59)	1.94 (1.53, 2.34)^∗^	*I* ^2^ = 0.0%, *p* = 0.846
	Asmare & Mekonnen, 2018	2.21 (1.57, 3.12)		
	Yismaw et al., 2019	1.68 (1.04, 2.72)		
	Mengistu et al., 2020	2.50 (1.81, 5.27)		
	Wosenu et al., 2017	1.80 (1.20, 2.60)		
Asphyxia	Gudayu et al., 2020	1.23 (0.79, 1.92)	2.03 (1.32, 2.75)^∗^	*I* ^2^ = 78.5%, *p* = 0.001
	Orsido et al., 2019	1.73 (1.25, 2.39)		
	Yismaw et al., 2019	2.18 (1.58, 3.03)		
	Wosenu et al., 2017	1.60 (1.05, 2.56)		
	Mengistu et al., 2020	4.84 (2.64, 5.90)		
Respiratory distress syndrome	Orsido et al., 2019	4.60 (3.10, 6.82)	2.49 (1.98, 3.00)^∗^	*I* ^2^ = 45.9%, *p* = 0.136
	Asmare & Mekonnen, 2018	2.27 (1.56, 3.28)		
	Alebel et al., 2020	2.50 (1.70, 3.70)		
	Wosenu et al., 2017	2.22 (1.50, 3.30)		
Hypothermia	Orsido et al., 2019	4.75 (3.33, 6.78)	3.12 (0.12, 6.13)	*I* ^2^ = 85.9%, *p* = 0.002
	Yismaw et al., 2019	1.68 (1.00, 2.82)		
Maternal illness	Asmare & Mekonnen, 2018	2.38 (1.63, 3.46)	1.91 (1.41, 2.41)^∗^	*I* ^2^ = 30.9%, *p* = 0.229
	Yismaw et al., 2019	1.71 (1.21, 2.40)		
Predictors of neonatal mortality in SSA using community-based studies				
Male gender	Limaso et al., 2020	2.61 (1.08, 6.29)	1.31 (1.22, 1.41)^∗^	*I* ^2^ = 0.0%, *p* = 0.493
	Wakgari & Wencheko, 2013	1.26 (1.06, 1.50)		
	Ezeh et al., 2014	1.29 (1.11, 1.51)		
	Dahiru, 2017	1.25 (1.10, 1.43)		
	Mekonen et al., 2013	1.42 (1.26, 1.59)		
Perceived small size	Desta et al., 2016	5.89 (2.44, 14.22)	1.85 (1.05, 2.65)^∗^	*I* ^2^ = 80.1%, *p* = 0.007
	Wakgari & Wencheko, 2013	1.38 (1.05, 1.82)		
	Ezeh et al., 2014	2.17 (1.82, 2.58)		
Multiple births	Limaso et al., 2020	3.48 (1.04, 11.68)	3.71 (1.72, 5.69)^∗^	*I* ^2^ = 86.9, *p* ≤ 0.001
	Desta et al., 2016	6.76 (4.64, 9.81)		
	Wakgari & Wencheko, 2013	3.73 (2.81, 4.94)		
	Dahiru, 2017	1.82 (1.43, 2.31)		
Rural residence	Ezeh et al., 2014	1.36 (1.11, 1.66)	1.30 (1.27, 1.32)^∗^	*I* ^2^ = 0.0%, *p* = 0.700
	Dahiru, 2015	1.30 (1.28, 1.33)		
	Mekonen et al., 2013	1.22 (1.03, 1.46)		
Home delivery	Limaso et al., 2020	2.55 (1.01, 6.42)	1.01 (0.84, 1.17)	*I* ^2^ = 20.4%, *p* = 0.262
	Ezeh et al., 2014	1.00 (0.84, 1.17)		
CS delivery	Ezeh et al., 2014	2.33 (1.54, 3.51)	1.58 (0.91, 2.20)	*I* ^2^ = 86.9, *p* ≤ 0.001
	Dahiru, 2017	2.09 (1.59, 2.74)		
	Dahiru, 2015	0.93 (0.81, 1.07)		
	Mekonen et al., 2013	1.26 (0.86, 1.85)		
Pregnancy complication	Wakgari & Wencheko, 2013 Dahiru, 2015	1.73 (1.27, 2.24)	1.35 (0.71, 1.99)	*I* ^2^ = 85.7%, *p* = 0.008
		1.07 (1.01, 1.13)		
ANC follow-up	Wakgari & Wencheko, 2013	0.72 (0.59, 0.89)	0.82 (0.66, 0.97)^∗^	*I* ^2^ = 75.9%, *p* = 0.042
	Dahiru, 2015	0.88 (0.85, 0.92)		
No PNC	Limaso et al., 2020	2.799 (1.16, 6.75)	—	—

BF: breastfeeding; CS: cesarean section; ANC: antenatal care; PNC: postnatal care.

## Data Availability

The data that support the review findings of this study are included in the manuscript, and the supporting files are submitted as additional files (Additional file 1 & Additional file 2) and PRISMA checklists and search strings.

## Abbreviations

ANC: Antenatal care
CI: Confidence interval
ENM: Early neonatal mortality
JBI: Juana Brigg's Institute
HH: Hazard ratio
LNM: Late neonatal mortality
IDR: Incidence density rate
NICU: Neonatal intensive care unit
NM: Neonatal mortality
NMR: Neonatal mortality rate
PHR: Pooled hazard ratio
SDGs: Sustainable development goals
SSA: Sub-Saharan Africa
RDS: Respiratory distress syndrome.

## Supplementary Materials

Supplementary 1 Extraction checklist of the included studies.

Supplementary 2 The critical appraisal of cohort studies.
