# Supplementary material for: Incidence Density Rate of Neonatal Mortality and Predictors in Sub-Saharan Africa: A Systematic Review and Meta-Analysis
Source: Int J Pediatr. 2020 Oct 15;2020:3894026. doi: 10.1155/2020/3894026 (PMC7586147; doi:10.1155/2020/3894026)
Supplement: Supplementary 1 — Extraction checklist of the included studies. [file 3894026.f1.docx]

| **Extraction checklist of the included studies** | | | | | | | | | | | | | |
| --- | --- | --- | --- | --- | --- | --- | --- | --- | --- | --- | --- | --- | --- |
| Sr. No | Author , Year | Country | Sample Size | Study Population | | Treatment Outcome | | Type | | Neonate days | IDR death (per 1000 neonate DAYS) | Factors CHR (95% CI) | Quality Score |
|  |  |  |  | preterm | term | Dead | Censored | ENM | LNM |  |  |  |  |
| 1 | Dessu et al, 2019 | ETHIOPIA | 332 | 37 | 30 | 67 | 265 | Na | Na | 2,121 | 31.6 | 1. APGAR SCORE<5 IN 5 MINUTE: 4.8 (3.61,9.87)  2. NO EBF IN 1 HOUR: 1.79(1.09,2.96)  3.BIRTH ORDER 2^ND^ TO 4^TH^ : 2.4 (1.194,4.934)  4. BIRTH ORDER >5: 9.4(4.71,18.68)  5.HISTOTY OF 2 TO 4 LIVE BIRTHS: 0.52(0.29,0.89)  6. HISTORY OF > LIVE BIRTHS: 0.78(0.33,1.83)  7. MATERNAL AGE <18 YEARS: 29.2(6.19,138.25)  8. CS delivery : 1.9(1.08,3.34)* |  |
| 2 | Gudayu et al, 2020 | ETHIOPIA | 504 | Na | Na | 87 | 417 | 73 | 14 | 2508 | 34.69 (28.11, 42.8) | 1.home delivery: 2.26 (1.04, 4.90)  2. ANC ; 1.85 (0.89, 3.84)  3. Sepsis: 1.95 (1.06, 3.59)  4. CRYING AT BIRTH: 2.74 (1.80, 4.18)  5..PRETERM BIRTH: 2.55 (1.59, 4.08)  6.ASPHYXIA: 1.23 (0.79, 1.92) |  |
| 3 | Mengesha et al, 2016 | ETHIOPIA | 1152 | Na | Na | 68 | 1084 | 50 | 18 | 27357.508 | 2.49 | 1.NO EBF: 21.63 (12.82,36.49)  2.NO NEONATAL COMPLICATION: 0.04 (0.024,0.068)  3. PROXIMITY>10KM: 4.64 (2.42,8.89)  4. NO DELIVERY complication : 0.23 (0.17,0.37)  5. LOW BIRTH WT: 11.49 (4.49,29.39)  6. CS Delivery: 1.17( 0.654,2.1) |  |
| 4 | Orsido et al, 2019 | ETHIOPIA | 964 | 84 | 75 | 159 | 804 | 132 | 27 | 5889 | 27 (23.1, 31.5) | 1.MULTIPLE BIRTH: 4.43 [3.24–6.07]  2. PRETERM: 3.60 [2.63–4.93]  3. NO ANC FOLLOW UP: 12.96 [7.86–21.39]  4. CS DELIVERY: 0.26 [0.15–0.46]  5. PNA: 1.73 [1.25–2.39]  6. HMD: 4.60 [3.10–6.82]  7.HYPOTHERMIA: 4.75 [3.33–6.78]  8. NEONATAL RESUSCITATION: 4.49 [3.10–6.49]  9.NO EBF IN 1 HR: 7.42 [4.73–11.64]  10. Female sex: 0.59 [0.42–0.84]  11.MAS: 0.74 [0.47–1.19] |  |
| 5 | Asmare & Mekonnen, 2018 | ETHIOPIA | 571 | 170 | - | 170 | 401 | 103 | 67 | 4354 | 39.04(33.59, 45.38) | 1.MALE: 1.70(1.24 , 2.33)  2. URBAN RESIDENCE: 0.52(1.43,2.61)  3. ANC: 0.38(0.28,0.53)  4.DM MOM: 2.38(1.63,3.46)  5. RDS: 2.27(1.56 ,3.28)  6.SEPIS : 2.21(1.57,3.12)  7.VERY PRETERM: 6.31(3.89 ,10.24)  8.1^ST^ MINUTE AGPAR<7: 3.22(1.92,5.39)  9.BF: 0.28(0.21,0.38)  10. 5^TH^ MINUTE APGAR<7: 3.83(2.70, 5.44) |  |
| 6 | Yismaw et al , 2019 | ETHIOPIA | 516 | 149 | - | 149 | - | 127 | 22 | 4527 | 32.9 | 1. HOME DELIVERY: 2.14 (1.08, 4.23)  2.MATERNAL ILLNESS: 1.71 (1.21, 2.40)  3. SINGLE PX: 1.71 (1.18, 2.46)  4.GA: 0.76 (0.70, 0.82)  5.SGA: 1.56 (1.10, 2.21)  6.NO CRY AFTER BIRTH: 0.41 (0.30, 0.57)  7. PNA: 2.18 (1.58 3.03)  8.KMC: 0.25 (0.12, 0.53)  9. LBW:1.36 (0.95, 1.96)  10.hypothermia: 1.68 (1.00, 2.82)  11. SEPSIS: 1.68 (1.04, 2.72)  12.HYPOGLYCEMIA: 2.10 (1.49, 2.95)  13. maternal complication: 1.55 (1.10, 2.18) |  |
| 7 | Alebel et al, 2020 | ETHIOPIA | 513 | 42 | 67 | 109 | 404 | 91 | 18 | 4223 | 25.8(21.4, 31.1) | 1. UNEMPLOYED MOM: 1.4 (0.9, 2.3)  2. NO ANC: 2.6 (1.5, 4.6)  3. EBF: 2.0 (1.3, 3.2)  4. RDS: 2.5 (1.7, 3.7)  5.SEVERE 1^ST^ MINUTE APGAR: 2.7 (1.5, 4.8) |  |
| 8 | Mengistu et al, 2020 | ETHIOPIA | 612 | 69 | 45 | 114 | 498 | 103 | 11 | 4177.8 | 34.47 | 1.AGE>35: 3.82 (2.03,4.35  2. UNABLE TO READ & WRITE: 2.54 (1.60,3.35)  3. MULTIPLE PX: 5.68 (2.95, 6.73)  4. NEONATAL SEPSIS: 2.50 (1.81,5.27)  5. HIV POSITIVE MOMS: 5.29 (2.24,5.36)  6. Female sex: 1.55 (0.96,2.01)  7. Delivery complication: 4.83 (2.47,6.30)  8. No cry at birth: 4.84 (2.64,5.90)  9. Rural residence: 1.98 (1.27,2.66) |  |
| 9 | Kahsay et al, 2020 | ETHIOPIA | 253 | - | - | 32 | 221 | 32 | - | 1425.32 | 22.45 | 1. MULTIPLE BIRTH:2.73 (1.18,6.32)  2. VLBW: 7.30 (3.41,15.63)  3. PRETERM: 5.83(2.52,13.49)  4. APGAR<7: 3.13(1.43,6.86)  5. Rural residence: 1.68 (0.84,3.36)  6. Female sex: 0.56 0.26,1.19 |  |
| 10 | Coulibaly et al, 2016 | BURKINA FASO | 341 | - | - | 18 | 323 | - | - | 9,326.4 | 1.93 (1.2–3.1) | 1. YOUNG MATERNAL AGE: 0.9(0.9, 1.0)  2. HISTORY OF INFANT DEATH: 4.6(1.8,11.5)  3. NOT IMMUNIZED: 4.5 (1.7,12.1)  4. PRETERM : 17.6 (6.9,44.8) |  |
| 11 | Musooko et al, 2014 | UGANDA | 341 | - | - | 37 | 304 | 37 | - | 1233.3 | 30 | - |  |
| 12 | Wosenu et al, 2017 | ETHIOPIA | 490 | - | - | 171 | 319 | 171 | - | - | - | 1.SEPSIS: 1.8 (1.2,2.6)  2. JAUNDICE: 2.4 (1.7,3.4)  3.PNA: 1.6 (1.05,2.56)  4.HDM: 1.9 (1.3,2.7)  5.RDS: 2.22 (1.5,3.3) |  |
| 13 | Limaso et al, 2020 | ETHIOPIA | 584 | - | 584 | 24 | - | 15 | 11 | 15894 | 1.51 | 1.NEONATAL COMPLICATION (5.39(2.31–12.59)  2. MALE SEX: 2.61 (1.08–6.29)  3. PERCEIVED SMALL SIZED: 5.89(2.44–14.22)  4. EBF>1HR: 2.81(1.12–7.09)  5.. NO POSTNATAL CARE: 2.799(1.16–6.75)  6.home delivery: 2.55(1.01–6.42)  7. Multiple birth: 3.48(1.04–11.68) |  |
| 14 | Gizaw et al, 2014 | ETHIOPIA | - | - | - | 1055 | - | 768 | 287 | 803,370 | 1.3(1.2, 1.4) | - |  |
| 15 | Desta et al, 2016 | ETHIOPIA | 7,367 | Na | Na | 209 | 7158 | 144 | 20 | 209,205 | 1 ( 0.87 - 1.15) | 1.TWIN BIRTH: 6.76 (4.64, 9.81)  2.PRETERM BIRTH: 15.53 (10.15, 23.77)  3.PREVEOUS SIBLING DEATH: 2.82 (1.85, 4.30)  4.NO PREVIOUS BIRTH: 2.23 (1.63, 3.04) |  |
| 16 | Wakgari &  Wencheko, 2013 | ETHIOPIA | 8,651 | - | - | 517 | - | 367 | 150 | - | - | 1. MULTIPLE BIRTHS:3.73 (2.81-4.94)  2. FIRST ORDER BIRTH: 1.68 (1.25-2.24)  3. MALE SEX: 1.26(1.06- 1.50)  4.BIRTH INTERVAL<2 YEARS:1.63(1.31-2.03)  5. VERY SMALL NEONATE: 1.38(1.05-1.82)  6. PREGNANCY COMPLICATIONS:1.73(1.27-2.24)  7.ANC VISIT: 0.72, 95% CI: 0.59-0.89)  8.BF WITH IN 1HR: 0.83 (0.59-0.99) |  |
| 17 | Ezeh et al, 2014 | NIGERIA | 27147 | - | - | 996 | 26,151 | - | - | - | - | 1.RURAL RESIDENCE: 1.36 (1.11, 1.66)  2.MOM AGE<20: 4.02 (2.99-5.40)  3.MALE NEONATE: 1.29 (1.11-1.51)  4.PERCIEVED SMALL SIZE: 2.17 (1.82-2.58)  5.CS DELIVERY: 2.33 (1.54-3.51)  6. home delivery: 1(0.84-1.17) |  |
| 18 | Dahiru, 2017 | NIGERIA | 244836 | - | - | 8176 | 236,660 | 8176 | - | - | - | 1.CS: 2.09 (1.59-2.74)  2.MULTILPE BIRTH: 1.82 (1.43-2.31)  3.MALE NEONATE: 1.25 (1.10-1.43) |  |
| 19 | Dahiru, 2015 | NIGERIA | 119024 | - | - | 3772 | 115,252 | 3772 | - | - | - | 1.RURAL RESIDENCE: 1.30 (1.28-1.33)  2.LARGE BW: 1.20 (1.13-1.27)  3.CS DELIVERY: 0.93 (0.81-1.07)  4. PX COMPLICATIONS: 1.07 (1.01-1.13)  5. ANC: 0.88 (0.85-0.92)  6. POSTNATAL CARE: 0.84 (0.77-0.92) |  |
| 20 | Mekonen et al, 2013 | ETHIOPIA | 32 042 | - | - | - | - | - | - | - | - | 1.Rural residence: (1.22 1.03 1.46)  2. Secondary edu. and above (0.65 0.49 0.87)  3.Male sex: (1.42 1.26 1.59)  4. maternal age (2.08 1.74 2.50)  5. CS delivery :( 1.26 0.86 1.85) |  |
