# Supplementary material for: Incidence Density Rate of Neonatal Mortality and Predictors in Sub-Saharan Africa: A Systematic Review and Meta-Analysis
Source: Int J Pediatr. 2020 Oct 15;2020:3894026. doi: 10.1155/2020/3894026 (PMC7586147; doi:10.1155/2020/3894026)
Supplement: Supplementary 2 — The critical appraisal of cohort studies. [file 3894026.f2.docx]

**The critical appraisal of cohort studies**

| Author, year | Q1 | | | | | Q2 | | | | Q3 | | | | Q4 | | | | Q5 | | | | Q6 | | | | Q7 | | | | Q8 | | | | Q9 | | | | Q10 | | | | Q11 | | | | Overall quality Result |
| --- | --- | --- | --- | --- | --- | --- | --- | --- | --- | --- | --- | --- | --- | --- | --- | --- | --- | --- | --- | --- | --- | --- | --- | --- | --- | --- | --- | --- | --- | --- | --- | --- | --- | --- | --- | --- | --- | --- | --- | --- | --- | --- | --- | --- | --- | --- |
|  | Y | | N | U | NA | Y | N | U | NA | Y | N | U | NA | Y | N | U | NA | Y | N | U | NA | Y | N | U | NA | Y | N | U | NA | Y | N | U | NA | Y | N | U | NA | Y | N | U | NA | Y | N | U | NA |  |
| Dessu et al, 2019 | | √ |  |  |  | √ |  |  |  | √ |  |  |  | √ |  |  |  | √ |  |  |  | √ |  |  |  | √ |  |  |  | √ |  |  |  |  |  | √ |  | √ |  |  |  | √ |  |  |  | 10/11 (90.9%) |
| Gudayu et al, 2020 | | √ |  |  |  | √ |  |  |  | √ |  |  |  | √ |  |  |  | √ |  |  |  | √ |  |  |  | √ |  |  |  | √ |  |  |  |  |  | √ |  | √ |  |  |  | √ |  |  |  | 10/11 (90.9%) |
| Mengesha et al, 2016 | | √ |  |  |  | √ |  |  |  | √ |  |  |  | √ |  |  |  | √ |  |  |  | √ |  |  |  | √ |  |  |  | √ |  |  |  |  |  | √ |  | √ |  |  |  | √ |  |  |  | 10/11 (90.9%) |
| Orsido et al, 2019 | | √ |  |  |  | √ |  |  |  | √ |  |  |  | √ |  |  |  | √ |  |  |  | √ |  |  |  | √ |  |  |  | √ |  |  |  |  |  | √ |  |  |  | √ |  | √ |  |  |  | 9/11 (81.82%) |
| Asmare & Mekonnen, 2018 | | √ |  |  |  | √ |  |  |  | √ |  |  |  |  |  | √ |  |  |  | √ |  | √ |  |  |  | √ |  |  |  | √ |  |  |  | √ |  |  |  |  |  | √ |  |  | √ |  |  | 7/11 (63.64%) |
| Yismaw et al , 2019 | | √ |  |  |  | √ |  |  |  | √ |  |  |  | √ |  |  |  | √ |  |  |  | √ |  |  |  | √ |  |  |  | √ |  |  |  |  |  | √ |  | √ |  |  |  | √ |  |  |  | 10/11 (90.9%) |
| Alebel et al, 2020 | | √ |  |  |  | √ |  |  |  | √ |  |  |  | √ |  |  |  | √ |  |  |  | √ |  |  |  | √ |  |  |  | √ |  |  |  |  |  | √ |  | √ |  |  |  | √ |  |  |  | 10/11 (90.9%) |
| Mengistu et al, 2020 | | √ |  |  |  | √ |  |  |  | √ |  |  |  | √ |  |  |  | √ |  |  |  | √ |  |  |  | √ |  |  |  | √ |  |  |  |  |  | √ |  |  |  | √ |  | √ |  |  |  | 9/11 (81.82%) |
| Kahsay et al, 2020 | | √ |  |  |  | √ |  |  |  | √ |  |  |  |  |  | √ |  |  |  | √ |  | √ |  |  |  | √ |  |  |  | √ |  |  |  | √ |  |  |  |  |  | √ |  |  | √ |  |  | 7/11 (63.64%) |
| Coulibaly et al, 2016 | | √ |  |  |  | √ |  |  |  | √ |  |  |  |  |  | √ |  |  |  | √ |  | √ |  |  |  | √ |  |  |  | √ |  |  |  |  |  | √ |  |  |  | √ |  |  | √ |  |  | 6/11 (54.54%) |
| Musooko et al, 2014 | | √ |  |  |  | √ |  |  |  | √ |  |  |  | √ |  |  |  | √ |  |  |  | √ |  |  |  | √ |  |  |  | √ |  |  |  |  |  | √ |  |  |  | √ |  |  | √ |  |  | 8/11 (72.72%) |
| Wosenu et al, 2017 | | √ |  |  |  | √ |  |  |  | √ |  |  |  |  |  | √ |  |  |  | √ |  | √ |  |  |  | √ |  |  |  | √ |  |  |  |  |  | √ |  |  |  | √ |  | √ |  |  |  | 7/11 (63.64%) |
| Limaso et al, 2020 | | √ |  |  |  | √ |  |  |  | √ |  |  |  | √ |  |  |  | √ |  |  |  | √ |  |  |  | √ |  |  |  | √ |  |  |  |  |  | √ |  | √ |  |  |  | √ |  |  |  | 10/11 (90.9%) |
| Gizaw et al, 2014 (14) | | √ |  |  |  | √ |  |  |  | √ |  |  |  | √ |  |  |  | √ |  |  |  | √ |  |  |  | √ |  |  |  | √ |  |  |  |  |  | √ |  |  |  | √ |  |  | √ |  |  | 8/11 (72.72%) |
| Desta et al, 2016 | | √ |  |  |  | √ |  |  |  | √ |  |  |  | √ |  |  |  | √ |  |  |  | √ |  |  |  | √ |  |  |  | √ |  |  |  |  |  | √ |  | √ |  |  |  | √ |  |  |  | 10/11 (90.9%) |
| Wakgari & Wencheko, 2013 | | √ |  |  |  | √ |  |  |  | √ |  |  |  |  |  | √ |  |  |  | √ |  | √ |  |  |  | √ |  |  |  | √ |  |  |  |  |  | √ |  |  |  | √ |  | √ |  |  |  | 7/11 (63.64%) |
| Ezeh et al, 2014 | | √ |  |  |  | √ |  |  |  | √ |  |  |  | √ |  |  |  | √ |  |  |  | √ |  |  |  | √ |  |  |  | √ |  |  |  |  |  | √ |  |  |  | √ |  |  | √ |  |  | 8/11 (72.72%) |
| Dahiru, 2017 | | √ |  |  |  | √ |  |  |  | √ |  |  |  |  |  | √ |  |  |  | √ |  | √ |  |  |  | √ |  |  |  | √ |  |  |  |  |  | √ |  |  |  | √ |  |  | √ |  |  | 6/11 (54.54%) |
| Dahiru, 2015 | | √ |  |  |  | √ |  |  |  | √ |  |  |  |  |  | √ |  |  |  | √ |  | √ |  |  |  | √ |  |  |  | √ |  |  |  |  |  | √ |  |  |  | √ |  | √ |  |  |  | 7/11 (63.64%) |
| Mekonen et al, 2013 | | √ |  |  |  | √ |  |  |  | √ |  |  |  |  |  | √ |  |  |  | √ |  | √ |  |  |  | √ |  |  |  | √ |  |  |  |  |  | √ |  |  |  | √ |  |  | √ |  |  | 6/11 (54.54%) |

****Y=yes, N=no, U=unclear, NA=not applicable, <60%=low, 60-80%=medium, >80%=high quality***
